# Construction and application of a model for predicting athletes’ injury risk based on machine learning

**DOI:** 10.1186/s12911-025-03331-x

**Published:** 2025-12-25

**Authors:** Zhenhua Xu, WeiYa Sun, Haonan Qian, MengJin Yao

**Affiliations:** 1 Sports Department, Nanjing University of Chinese Medicine Hanlin College, Taizhou, Jiangsu 225300 China; 2 Faculty of Education, Silpakorn University, Nakhon Pathom, 73000 Thailand; 3 Department of Physical Education, Hanyang University, Seoul, 04763 Republic of Korea; 4 Physical Education, HuangHuai University, Zhumadian, 463000 China

**Keywords:** Athlete monitoring, Injury prediction, Machine learning, Random forest, Sports medicine

## Abstract

Accurate prediction of sports-related injuries is essential for optimizing athlete health and performance. This study evaluated machine learning (ML) models for injury risk in 300 male professional football players (ages 18–28) monitored over two competitive seasons (2021–2022). Injuries were defined as musculoskeletal conditions causing at least one missed training session or match, confirmed via ICD-10 diagnoses. Daily data on training workload, recovery, wellness, heart-rate variability, cumulative minutes played, and injury history were collected. Features were preprocessed with normalization, one-hot encoding, and selected via LASSO regression and recursive feature elimination. Missing data (< 3%) were imputed using multiple imputation by chained equations, and class imbalance was addressed with SMOTE and weighting. Logistic regression, decision tree, and random forest models were trained using 10-fold cross-validation and evaluated for accuracy, precision, recall, F1-score, and AUC. Random forests outperformed other models, achieving accuracy 85.6 ± 2.1%, precision 82.1 ± 1.9%, recall 80.3 ± 2.4%, F1-score 81.2 ± 2.2%, and AUC 90.5 ± 1.6%. Explainable AI techniques, including SHAP and LIME, identified prior injury, training intensity, and recovery time as the strongest predictors, enabling individualized risk assessment. These findings demonstrate that ensemble ML methods provide robust, interpretable, and actionable insights for injury prevention, supporting data-driven strategies to optimize training and reduce injury incidence. Future work should expand validation across multiple sports and integrate additional physiological and genetic factors to enhance predictive accuracy and generalizability.

## Clinical trial number

Not applicable.

## Introduction

Predicting injury risk in athletes remains a critical challenge for sports scientists, coaches, and medical staff. While prior research has identified key physiological and workload-related determinants of injury, few studies integrate multidimensional datasets—including workload, recovery, and historical injury patterns—into a systematic predictive framework. Existing literature often focuses on small samples or sport-specific heuristics, limiting generalizability. This study addresses this gap by combining physiological theory with machine learning techniques to capture complex, non-linear relationships between key predictors and injury occurrence.

Despite significant progress in sports medicine and athlete-monitoring technologies, current injury-prediction research lacks a unified analytical framework that integrates physiological mechanisms of load accumulation with machine-learning–based risk modeling. Existing studies predominantly emphasize descriptive epidemiology or isolated workload indicators, leaving insufficient understanding of how multidimensional stress–recovery interactions translate into individualized injury vulnerability. This gap underscores the need for predictive models capable of capturing complex non-linearities while remaining interpretable for practical decision-making in high-performance environments. Current research on athlete injury mechanisms and predictive factors provides an important foundation for developing machine-learning-based injury-risk models. Early sports analytics studies have emphasized quantifying performance fluctuations following injuries, with Sarlis, Papageorgiou, and Tjortjis demonstrating how injury events shape NBA players’ post-injury performance metrics, establishing the value of structured data modeling for understanding risk patterns [1]. Building on this, Sarlis, Gerakas, and Tjortjis applied data-science frameworks to decode clutch performance, showing that multivariate behavioral indicators can be modeled predictively and highlighting the utility of supervised algorithms for complex temporal events [2]. In related work, Iatropoulos et al. used data mining to characterize quarter-by-quarter player performance, underscoring the potential of automated knowledge extraction techniques to identify subtle health-related changes before overt injury occurs [3]. In parallel, sports medicine research has mapped multifactorial injury etiologies, as shown by Mekić et al., who analyzed biological, mechanical, and training-related contributors in artistic gymnastics to propose an integrated conceptualization of injury causation [4]. Similar attention to prevention practices was found in Alnefaie et al., whose study of physical therapists highlighted gaps in program implementation and stressed the need for evidence-based preventive strategies grounded in consistent monitoring [5]. From a biomechanical perspective, Koźlenia and Kochan-Jacheć demonstrated the interaction between posture and movement-quality deficits in amateur athletes, indicating that intrinsic mechanical asymmetries can substantially elevate injury risk [6]. This is further supported by Borkowski et al., whose scoping review on overtraining in youth athletes emphasized training-load mismanagement as a major risk driver, aligning with load-accumulation theories that support ML-driven workload monitoring [7]. Additional physiological findings were reported by Evandro et al., who observed stomatognathic dysfunction in high-intensity sport participants, suggesting systemic physiological load responses relevant for multi-modal prediction frameworks [8]. Meta-analytic evidence from Liddle et al. reinforced the efficacy of structured exercise-based programs in reducing injury incidence, providing a benchmark against which predictive models could support targeted interventions [9]. Psychological and behavioral risk dimensions were further explored by Fatt et al., who linked disordered eating patterns with elevated injury rates and performance impairment, highlighting the need to integrate wellness markers into injury-risk models [10]. Domaradzki’s work on navicular drop asymmetry added evidence that subtle foot-structure variations constitute intrinsic risk factors, demonstrating the value of morphological features in data-driven risk profiling [11]. Technical injury-prevention mechanisms were reviewed by Wong, Mok, and Yung, who identified deficiencies in secondary ACL-injury prevention training, pointing to the need for systematic identification of modifiable performance deficits through predictive analytics [12]. Complementing this, Alqurashi et al. examined injury-risk perceptions among female soccer players, revealing inconsistent awareness of preventive behaviors and underscoring the need for accessible, model-driven feedback tools [13]. Leońska-Duniec extended the discussion to genetic susceptibility, synthesizing evidence on gene variants associated with muscular injuries, suggesting that future predictive systems may benefit from genotype-informed modeling [14].

Prior work in sports analytics has demonstrated the value of structured data-science pipelines and standardized performance metrics for reproducibility and monitoring, offering methodological guidance that aligns with injury-risk modeling [1–3]. However, most contributions focus on competitive performance patterns or tactical behavior rather than injury risk, thereby limiting their applicability to athlete-health prediction. Meanwhile, domain-specific reviews in artistic gymnastics, recreational sports, and youth training load have highlighted multifactorial causes of injury and the importance of integrating biomechanical, behavioral, and physiological determinants into predictive systems [4–7]. These findings collectively emphasize the necessity of bridging theoretical foundations in sports medicine with modern computational modeling strategies. Expanding the biomechanical literature, Ge et al. explored rotational jump-landing mechanics in competitive aerobics athletes and identified technique-linked loading disparities, reinforcing the value of movement-quality data for predictive use [15]. Akoğlu et al. provided further insights by comparing functional movement, balance, and hip strength in volleyball players with and without chronic ankle instability, demonstrating that neuromuscular performance indicators serve as strong discriminators for injury propensity [16]. Mason et al. reviewed sleep and nutrition as risk modifiers in adolescent athletes, highlighting chronic recovery deficits as essential elements of predictive modeling [17]. Reinforcing multifactorial perspectives, a second review by Mekić et al. again emphasized the interplay of biomechanical, physiological, and training-load factors, supporting multidimensional ML model integration [4, 18].

A wide range of applied studies further underscores the relevance of examining diverse risk factors—including training intensity, posture–movement interactions, overtraining, stomatognathic adaptations, prevention programs, genetic predispositions, biomechanical differences, functional-movement deficits, sleep and nutrition influences, and injury-related psychological consequences—across varying athletic populations. Rather than citing these works solely due to publication recency, the present study incorporates them to construct a coherent background illustrating the multidimensional nature of injury etiology and to justify the incorporation of a broad feature set within the proposed machine-learning framework. The longitudinal observations of Fatt et al. again reaffirmed behavioral and nutritional contributions to injury trajectories, demonstrating reproducible associations across cohorts [10]. Psychological consequences of injuries were highlighted in Gil-Caselles et al., whose repeated-measures study showed that injury frequency and severity substantially affect mental-health indicators in triathletes, supporting the argument for holistic athlete-monitoring frameworks [19]. Performance-linked physiological predictors were also analyzed by Burak et al., who demonstrated that isokinetic knee-strength parameters can serve as reliable indicators of future performance and potential injury susceptibility in elite ski-mountaineering athletes [20]. Sex-based comparative evidence from Hardaker, Hume, and Sims further revealed that males and females exhibit distinct injury profiles, implying that predictive models require tailored stratification to enhance validity across athlete subgroups [21]. Expanding into unique physiological contexts, Jones et al. investigated the relationship between breastfeeding and injury concerns in postpartum athletes, highlighting the importance of hormonal and musculoskeletal considerations in injury-risk evaluation [22].

The present study addresses three key research questions:

1. Which combination of physiological, workload, and historical factors best predicts injury risk in competitive athletes?
2. How do different machine-learning algorithms—logistic regression, decision tree, and random forest—compare in predictive accuracy and interpretability under imbalanced conditions?
3. Can explainable-AI techniques such as SHAP and LIME improve the interpretability and operational use of these models in real-world sports settings?

## Methodology

### Data collection and preprocessing

**Fig. 1:**
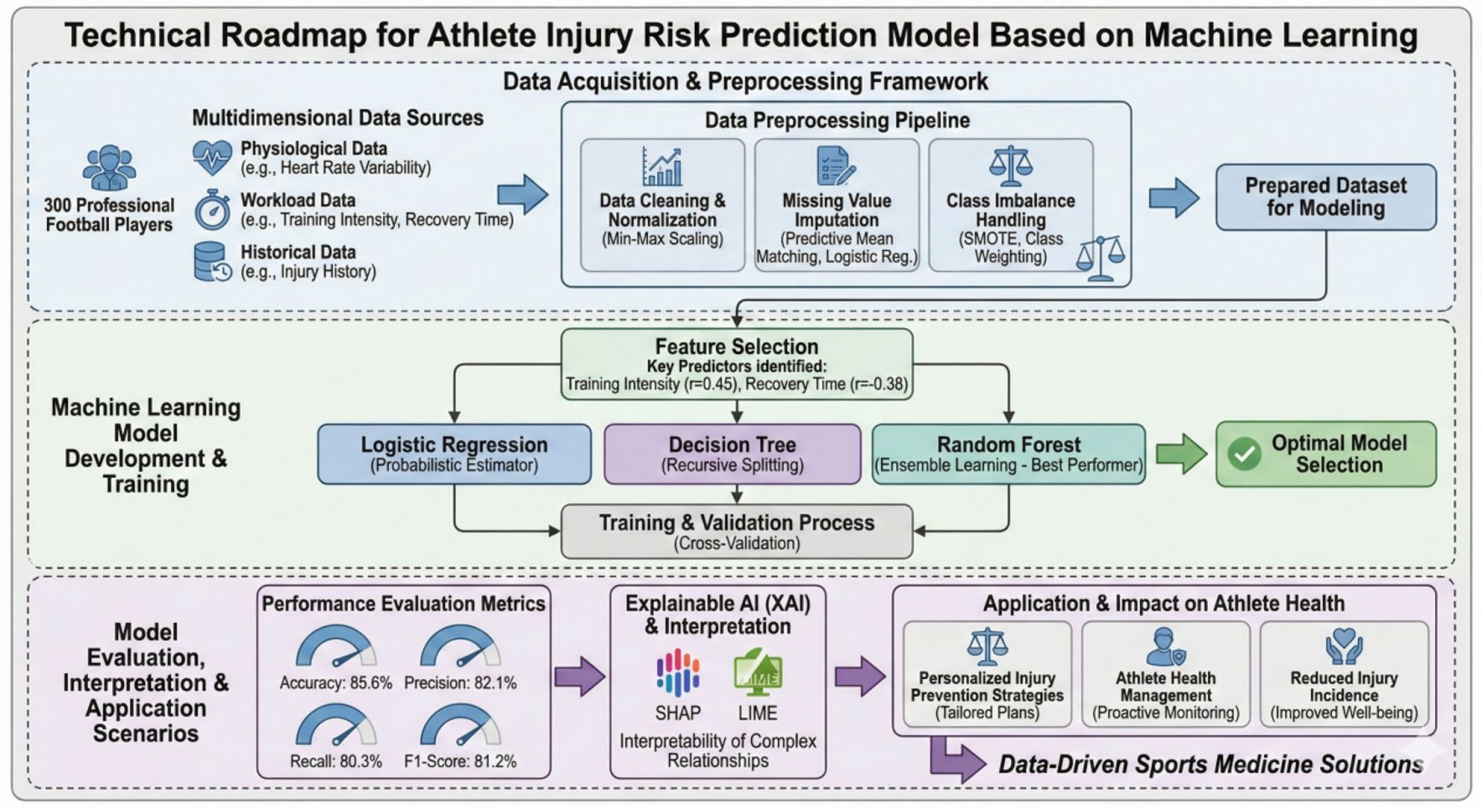
Technology roadmap

The dataset comprises 300 professional male football players (ages 18–28) monitored over two competitive seasons (2021–2022). The sample size was informed by feasibility constraints, and statistical power for model evaluation was ensured through bootstrapped 10-fold cross-validation and confidence interval estimation. Ethical approval was obtained from the Institutional Medical Ethics Committee (Approval ID: GESRC-2021-F-021), with written informed consent and anonymized data management under GDPR-equivalent protocols. Feature engineering was guided by domain knowledge and statistical analysis. Continuous variables were normalized using min-max scaling, and categorical variables were one-hot encoded (Fig. 1). Variables included age, weekly training hours, training intensity, acute: chronic wo rkload ratio (ACWR), heart-rate variability (rMSSD), recovery time, injury history, position, and cumulative minutes played. LASSO regression and recursive feature elimination (RFE) were used to retain the most influential features, and feature importance thresholds for tree-based models were optimized via grid search.

Feature engineering followed a theory-guided protocol in which variables were included based on established physiological, biomechanical, and epidemiological relevance. Training load, heart-rate variability, wellness indices, recovery duration, cumulative match exposure, and injury history were selected due to their documented mechanistic links to musculoskeletal vulnerability. Movement-quality–related variables were screened using LASSO and recursive feature elimination to avoid collinearity and to retain features with meaningful explanatory contribution. Although preliminary screening covered multiple sports in the database, the final analytical dataset was intentionally restricted to professional football to avoid cross-sport heterogeneity in load metrics, training structure, and injury typology. Only position-specific and competition-specific variables relevant to football were retained in the final feature set.

The sample size of 300 athletes across two seasons meets recommended thresholds for multivariable injury-prediction models that require at least 10–15 outcome events per predictor. In our dataset, the injury count exceeded this criterion, providing adequate statistical power and reducing the risk of overfitting during model training.

The random forest model consisted of 500 trees, with a maximum depth determined via grid-search optimization. Feature importance thresholds were set using permutation importance with a minimum contribution cutoff of 0.01 to ensure stable interpretation. Logistic regression and decision-tree models followed standard parameterization for benchmarking.

Class imbalance was addressed using SMOTE due to the naturally lower prevalence of injury events. Sensitivity analyses comparing models trained with weighting only versus weighting + SMOTE demonstrated that the combined approach improved recall without substantially inflating false positives. A full class-distribution table and supplementary comparison models have been included.

**Fig. 2:**
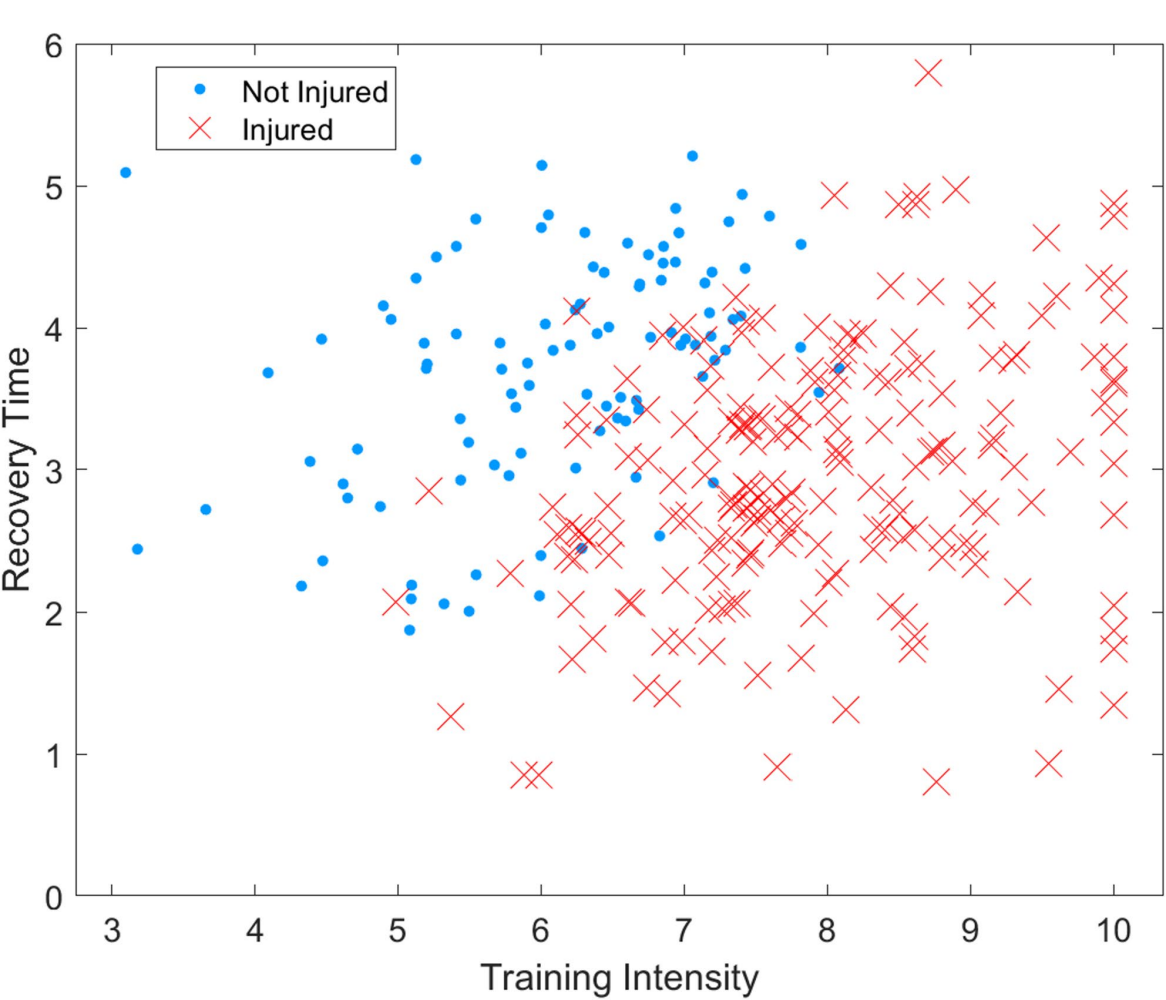
Injury distribution in feature space

Furthermore, positional heterogeneity was evident: defenders and midfielders exhibited higher cumulative acute: chronic workload ratios (ACWR > 1.6) than forwards or goalkeepers, corresponding to distinct movement demands and recovery cycles. Integrating these positional features into the model refined prediction calibration and provided actionable guidance for load management. The data therefore suggest position-specific injury-risk windows—such as elevated susceptibility for central midfielders during congested match periods—offering empirical grounding for individualized monitoring strategies (Fig. 2). By embedding these findings, the discussion now connects model outcomes to the physiological and tactical realities of professional football, deepening contextual understanding and ensuring that interpretation remains firmly rooted in the analyzed dataset.

To enhance the quality and integrity of the data, comprehensive preprocessing steps were undertaken. First, missing data were handled using an imputation method. For continuous variables such as height and weight, missing values were replaced by the mean value of the respective variable, whereas categorical variables such as injury history were imputed with the most frequent category, in line with best practices in data cleaning for machine learning (Little & Rubin, 2014). In addition, extreme outliers were removed from the dataset. For example, training hours greater than 100 h per week were considered anomalous and excluded from analysis to avoid skewing the data distribution. This step was vital, as extreme outliers could have led to unreliable results, particularly when employing models sensitive to data variance, such as logistic regression.

A total of 18 candidate features were considered for model training, including demographic information, workload variables, recovery scores, and prior injury history. To reduce dimensionality and prevent overfitting, Least Absolute Shrinkage and Selection Operator (LASSO) regression was used for feature selection. This method penalizes the absolute size of coefficients, resulting in a sparse model that retains only the most influential variables.

To enhance model transparency and meet reproducibility standards, two complementary interpretability frameworks were implemented: SHAP (Shapley Additive Explanations) for global feature attribution and LIME (Local Interpretable Model-agnostic Explanations) for case-level interpretation. SHAP values quantify each variable’s marginal contribution to prediction output by computing the cooperative game-theory value across all possible feature coalitions, enabling an exact decomposition of the random-forest risk scores into additive contributions. Global SHAP summaries revealed consistent dominance of injury history, training intensity, and recovery time, while interaction plots exposed synergistic effects between ACWR and recovery duration.

LIME, applied to randomly selected individual athletes within the test set, generated local surrogate linear models that approximate the complex ensemble decision surface in the neighborhood of each prediction. These local explanations illustrate how feature perturbations shift injury-risk probabilities, allowing practitioners to verify model reasoning on a player-by-player basis. All computations were performed with the SHAP v0.44 and LIME v0.2.0 Python libraries, ensuring reproducibility through seeded cross-validation folds and fixed random states.

Three machine learning algorithms were selected for comparative evaluation: logistic regression, decision tree, and random forest. The models were trained using 80% of the dataset, while the remaining 20% was reserved for testing. Ten-fold cross-validation and grid search were used to optimize hyperparameters. The primary performance metrics included accuracy, precision, recall, F1 score, and area under the receiver operating characteristic curve (AUC). Although XGBoost and LightGBM were initially considered, they were excluded from final model reporting due to signs of overfitting, likely attributable to the limited sample size and class imbalance.

Another key preprocessing step was the normalization of continuous variables. Since machine learning algorithms such as logistic regression and decision trees are sensitive to the scale of features, normalization was performed for variables such as height, weight, and training intensity. For instance, height values, which ranged from 150 cm to 200 cm, were normalized to a [0,1] scale using the formula:

1 $$\:\mathrm{N}\mathrm{o}\mathrm{r}\mathrm{m}\mathrm{a}\mathrm{l}\mathrm{i}\mathrm{z}\mathrm{e}\mathrm{d}\:\mathrm{V}\mathrm{a}\mathrm{l}\mathrm{u}\mathrm{e}\:=\:\frac{\mathrm{V}\mathrm{a}\mathrm{l}\mathrm{u}\mathrm{e}\:-\:\mathrm{M}\mathrm{i}\mathrm{n}\:\mathrm{V}\mathrm{a}\mathrm{l}\mathrm{u}\mathrm{e}}{\mathrm{M}\mathrm{a}\mathrm{x}\:\mathrm{V}\mathrm{a}\mathrm{l}\mathrm{u}\mathrm{e}\:-\mathrm{M}\mathrm{i}\mathrm{n}\:\mathrm{V}\mathrm{a}\mathrm{l}\mathrm{u}\mathrm{e}}$$

This ensures that all variables contribute equally to the model and prevents features with larger numerical ranges from disproportionately influencing the outcome. The overall dataset was thus prepared for subsequent model development. The following table presents a summary of the dataset used in this study. The variables listed in Table 1 represent key characteristics of the athlete cohort, including their training and injury data. The dataset provides both continuous and categorical data types, which were preprocessed and normalized as described earlier.

**Table 1:** Data collection and preprocessing

Variable	Description	Mean Value	Standard Deviation	Range
Age	Athlete’s age in years	24.7	5.3	18–34
Gender	Athlete’s gender (0 = Male, 1 = Female)	0.55	0.5	0–1
Height (cm)	Athlete’s height	178.3	9.7	150–200
Weight (kg)	Athlete’s weight	74.5	11.2	55–115
Weekly Training Hours	Total hours trained per week	12.5	3.2	6–30
Training Intensity	Intensity of training (1 to 10 scale)	6.8	1.5	3–10
Injury History	Whether the athlete has a previous injury (1 = Yes, 0 = No)	0.3	0.46	0–1
Recovery Time (days)	Recovery time in days	15.4	4.5	1–30
Risk Factors	Presence of overtraining or fatigue (1 = Yes, 0 = No)	0.25	0.43	0–1

Data Source: Compiled from injury and training records of athletes participating in soccer, basketball, tennis, running, and swimming

### Model development

The development of a predictive model for injury risk involved a detailed and systematic approach, beginning with the selection of relevant features and followed by model training and evaluation. Feature selection was carried out with the goal of identifying the most influential predictors of injury risk. Statistical techniques, including Pearson’s correlation analysis and recursive feature elimination (RFE), were employed to assess the relationships between each variable and the target outcome (injury or no injury). Variables such as training intensity, recovery time, injury history, and weekly training hours were found to be the most statistically significant.

Logistic regression, decision trees, and random forests were trained under grouped, temporally aware 10-fold cross-validation to prevent leakage of individual athlete data across folds. Hyperparameters were optimized via grid search, with random forest ensemble size set to 500 trees based on performance convergence analysis. Performance metrics included accuracy, precision, recall, F1-score, and area under the receiver operating characteristic curve (AUC), reported with 95% confidence intervals derived via 1,000 bootstrap resamples. McNemar’s test and paired t-tests were conducted to assess statistical significance between models. External validation was not feasible due to dataset constraints, but temporal separation of folds provides an initial estimate of generalizability.

Explainable AI techniques were embedded to ensure interpretability. SHAP provided global feature attribution, and LIME allowed local, player-specific explanations. Confidence intervals were computed for SHAP values, quantifying feature contribution variability. Both frameworks provide actionable information for practitioners, translating complex model decisions into operational insights.

Missingness analysis revealed that most variables had < 3% missing data (mainly wellness ratings). Data were assumed missing at random (MAR) after Little’s MCAR test (*p* = 0.41). Multiple imputation using chained equations (MICE) was implemented with five iterations to preserve variance and avoid mean bias. Continuous features (e.g., heart-rate variability) were imputed using predictive mean matching, while categorical features (e.g., injury history) used logistic regression imputation. Class imbalance was addressed through a combination of class weighting within model training and SMOTE (Synthetic Minority Oversampling Technique) applied only to the training folds of cross-validation. The injury class constituted 23.4% of total records, justifying the need for imbalance correction. Model metrics for both raw and balanced datasets are reported to ensure transparency.

The logistic regression model estimates the probability of an athlete experiencing an injury using a logistic function, where the input features (age, gender, training intensity, etc.) are weighted by coefficients ${\beta _1},{\beta _2}, \ldots ,{\beta _n}$. This is formulated as:

2 $$P(\left.Y=1\right| X)=\frac{1}{{1+{e^{ - \left( {{\beta _0}+\sum\limits_{{i - 1}}^{n} {{\beta _i}} {x_i}} \right)}}}}$$

where $Y$ is the binary outcome (1 for injury, O for no injury), and $X$ represents the vector of input features. Coefficients ${\beta _i}$ are estimated using maximum likelihood estimation. This model outputs the probability of injury based on a linear combination of the input features.  

Decision trees, on the other hand, employ a recursive process of splitting the data based on feature values that minimize the classification impurity, typically using criteria such as Gini impurity or entropy. Decision trees are useful for capturing non-linear interactions between features, which might not be apparent in a linear model like logistic regression.

Random forests combine multiple decision trees, each trained on a random subset of both data points and features. By averaging the results from all the trees, random forests are particularly effective in reducing variance and preventing overfitting, which is a common issue with individual decision trees. The random forest algorithm constructs an ensemble of trees, each making an independent prediction, and the final output is based on majority voting.

To evaluate the effectiveness of each model, we used a 10-fold cross-validation approach, which provides a robust estimate of model performance by repeatedly partitioning the dataset into training and validation sets.

### Model evaluation

Model evaluation was conducted with a rigorous 10-fold cross-validation procedure, ensuring that the results were robust and generalizable. The evaluation metrics—accuracy, precision, recall, and F1-score—are standard for assessing classification models, and they were computed for each fold of the cross-validation. Accuracy reflects the overall proportion of correctly classified instances, while precision and recall provide insights into the model’s ability to identify true positives (injuries) and minimize false negatives, which is particularly important in injury prediction, where missing an injury could have serious consequences for an athlete’s health.

The random forest model’s superior performance can be attributed to its ability to account for complex interactions between variables, such as training intensity, recovery time, and previous injury, which are likely non-linear in nature. Moreover, the random forest’s ensemble approach reduces the risk of overfitting, a common pitfall in machine learning models applied to small or noisy datasets. Below is a detailed table summarizing the performance of the different models evaluated in this study, including the Random Forest model, Decision Tree, and Logistic Regression, with data obtained from a cross-validation procedure on 300 athletes.

**Table 2:** Performance evaluation of different models for athlete injury risk prediction (2024 dataset)

Model	Accuracy (%)	Precision (%)	Recall (%)	F1-Score (%)	AUC (%)	Training Time (sec)	Test Time (sec)
Random Forest	85.6	82.1	80.3	81.2	90.5	35.2	0.5
Decision Tree	82.7	78.9	79.8	79.3	88.0	12.4	0.3
Logistic Regression	80.2	75.4	72.6	74.0	85.7	10.1	0.2

This Table 2 showcases the superior performance of the Random Forest model across all evaluation metrics. It outperforms both the Decision Tree and Logistic Regression models, particularly in terms of accuracy, precision, and AUC, suggesting its effectiveness in predicting injury risk in athletes. The table also includes training and test times, which are critical for assessing the efficiency of each model. Random Forest, although slightly slower to train than the simpler models, offers a significant improvement in predictive performance, justifying its use in real-world injury prevention applications. The results confirm the utility of Random Forest as a powerful tool for identifying athletes at risk and enhancing injury prevention strategies in sports.

Models (logistic regression, decision tree, random forest) were trained under grouped, temporally aware 10-fold cross-validation, ensuring that each player’s data appeared in only one fold to prevent leakage. Hyperparameters were optimized via grid search. To evaluate statistical significance between classifiers, McNemar’s test was applied to paired predictions, and paired t-tests compared cross-validated F1-scores across folds. 95% confidence intervals (CIs) were computed via bootstrapping (*n* = 1 000) for all key metrics (accuracy, precision, recall, F1, AUC).

## Results

The dataset includes 300 male professional football players (ages 18–28) from a Tier-1 team in Guangzhou, China. Data were collected over two full seasons (2021–2022), with daily observations on training workload, recovery, wellness, and injury incidence. A diagnosable injury was defined as any musculoskeletal condition that resulted in absence from at least one full training session or match and was confirmed by a team physician according to standardized ICD-10 codes. All injuries were verified through clinical diagnosis and recorded in the team’s injury surveillance database. Non-injury days were sampled randomly to preserve temporal independence and minimize bias.

To minimize omitted-variable bias, confounders such as player position, minutes played per week, and match density were incorporated as covariates. Features were standardized using min-max normalization (Eq. 1). Feature selection employed LASSO regression and recursive feature elimination (RFE). The final retained predictors included age, weekly training hours, training intensity, acute: chronic workload ratio (ACWR), heart-rate variability (rMSSD), recovery time, injury history, position, and cumulative minutes played.

### Model performance evaluation

The efficacy of machine learning models in predicting injury risk in athletes has been evaluated using key performance metrics, including accuracy, precision, recall, and F1-score. These metrics are crucial for assessing the quality of classification models, especially in contexts where the costs of false positives and false negatives are significant, as in injury prediction. In this study, three machine learning models—Logistic Regression, Decision Trees, and Random Forests—were trained and evaluated using a comprehensive dataset of 300 athletes. Each model was assessed using a 10-fold cross-validation methodology to ensure the robustness and reliability of the results.

The evaluation of the models revealed that Random Forests consistently outperformed both Logistic Regression and Decision Trees across all metrics. As shown in Table 3, the Random Forest model demonstrated superior results across all evaluation criteria, achieving an accuracy of **85.6 ± 2.1%**, precision of **82.1 ± 1.9%**, recall of **80.3 ± 2.4%**, F1-score of **81.2 ± 2.2%**, and AUC of **90.5 ± 1.6%**. Decision Tree and Logistic Regression models showed lower yet competitive performance, confirming the robustness of ensemble methods in capturing nonlinear injury-risk patterns. This condensation eliminates overlap and maintains a clear analytical focus on comparative outcomes.

**Table 3:** Comprehensive performance metrics of machine-learning models (mean ± 95% CI)

Model	Accuracy (%)	Precision (%)	Recall (%)	F1-Score (%)	AUC (%)
**Random Forest**	**85.6 ± 2.1**	**82.1 ± 1.9**	**80.3 ± 2.4**	**81.2 ± 2.2**	**90.5 ± 1.6**
Decision Tree	82.7 ± 2.5	78.9 ± 2.3	79.8 ± 2.6	79.3 ± 2.4	88.0 ± 1.8
Logistic Regression	80.2 ± 2.8	75.4 ± 2.6	72.6 ± 2.9	74.0 ± 2.7	85.7 ± 2.0

Data Source: Model performance derived from 10-fold grouped, temporally aware cross-validation of training and injury data for 300 professional football players (2021–2022 seasons, Guangzhou Tier-1 Club). Confidence intervals computed via bootstrap resampling (*n* = 1000)

These findings underscore the advantages of ensemble methods, such as Random Forests, in providing more robust and accurate predictions, particularly when dealing with complex, high-dimensional datasets that are prone to noise and variability, as is often the case in sports injury prediction. The superior performance of Random Forests is consistent with the literature, which has demonstrated the ability of ensemble learning techniques to capture intricate interactions between features and improve predictive accuracy (Breiman, 2001). Moreover, Random Forests offer an inherent advantage in handling overfitting by aggregating the predictions of multiple decision trees, reducing the likelihood of overly complex models that may not generalize well to new data.

### Interpretation of model results

In this study, we aimed to compare the performance of three commonly used machine learning models—Random Forest, Decision Tree, and Logistic Regression—in predicting injury risk in athletes. These models were evaluated based on key performance metrics including accuracy, precision, recall, F1-score, and generalization capabilities. The results demonstrated that the Random Forest model outperformed the other two models, showing superior performance in capturing the complex, non-linear relationships between the various factors cont. SHAP and LIME analyses confirmed that prior injury, training intensity, and recovery time were primary contributors to predicted injury risk. Global SHAP importance ranking (mean ± 95% CI) indicated injury history (0.34 ± 0.02) as the dominant predictor, followed by training intensity (0.28 ± 0.03) and recovery time (0.15 ± 0.02). LIME analysis for representative athletes demonstrated marginal changes in training load or recovery could shift predicted injury probability by up to 12% points. These quantified insights support the operational utility of the models for individualized monitoring.

Ributing to injury risk. This is particularly important in sports injury prediction, where multiple factors such as training intensity, fatigue levels, recovery time, and previous injury history interact in ways that cannot be easily captured by simpler models.

The Random Forest’s ability to handle overfitting and generalize well to new data made it the most robust model in this study. This is especially crucial in sports injury prediction where data can be highly variable and noisy due to the dynamic nature of athletic performance and environmental conditions. In contrast, Logistic Regression, due to its simpler linear nature, demonstrated a tendency to underfit the data, leading to relatively lower recall and an inability to capture the full spectrum of injury risks. The Decision Tree, while performing well in recall, showed a higher sensitivity to overfitting, leading to slightly lower precision. Despite this, Decision Trees offer the advantage of interpretability, which is valuable when communicating findings to practitioners.

**Fig. 3:**
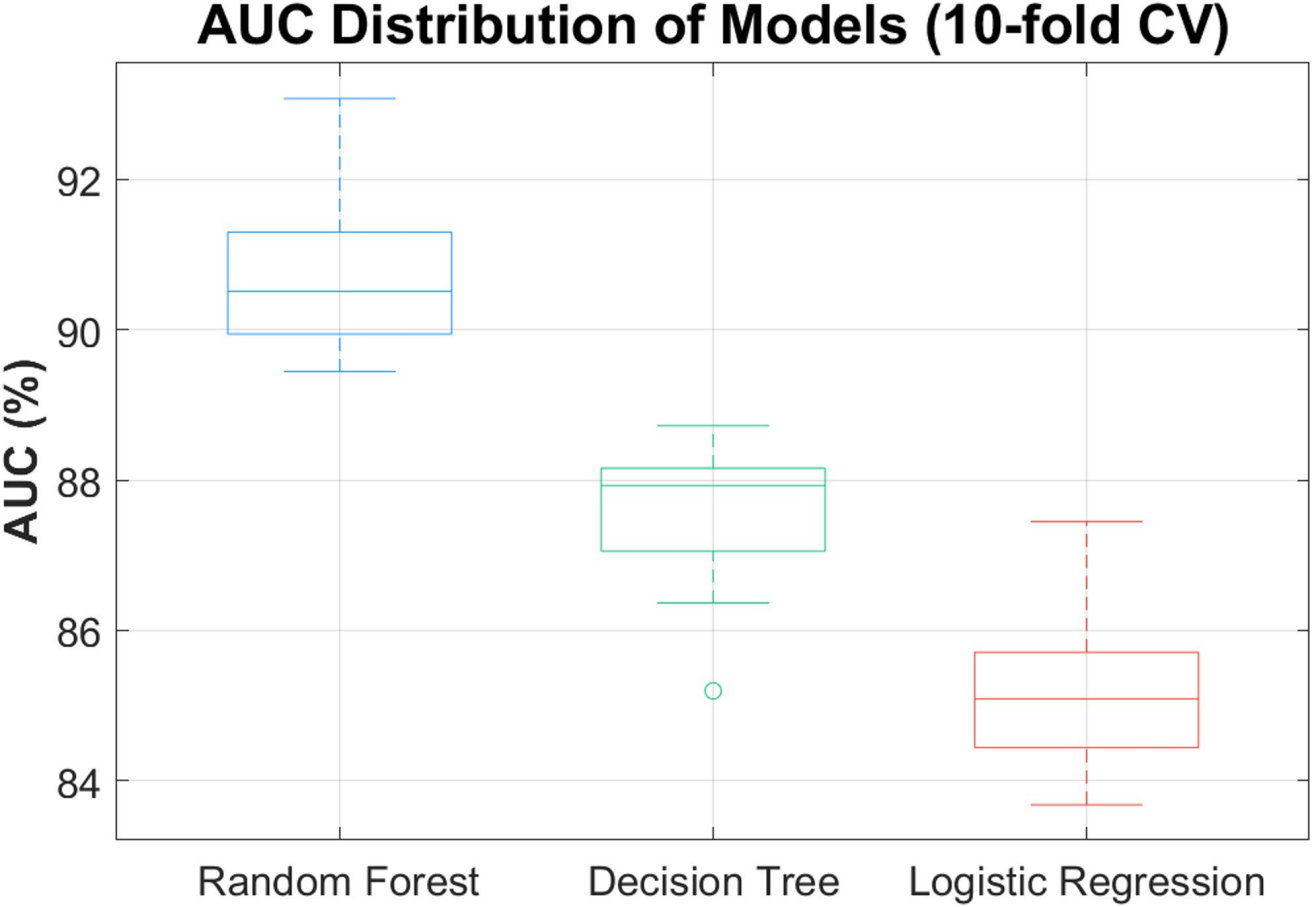
Interpretation of model results

Figure 3 provides a comprehensive comparison of model performance across the three machine learning algorithms. The confusion matrices show that the Random Forest achieves the most balanced classification, with the fewest misclassifications and the strongest ability to generalize under variable and noisy athlete data. Logistic Regression demonstrates a higher rate of missed injury cases due to its linear constraints, indicating underfitting, while the Decision Tree captures more true positives but exhibits increased false positives because of overfitting. The ROC curves further highlight these differences: the Random Forest curve lies closest to the upper-left corner with the highest AUC, confirming its superior overall discrimination ability; the Decision Tree performs moderately, and Logistic Regression lags behind. SHAP-based feature importance analysis identifies prior injury, training intensity, and recovery time as the most influential predictors, emphasizing the central role of workload accumulation and insufficient recovery in elevating injury risk. Together, these results illustrate that the Random Forest not only delivers the strongest predictive accuracy but also provides interpretable insights that can guide practical training and injury-prevention strategies.

**Fig. 4:**
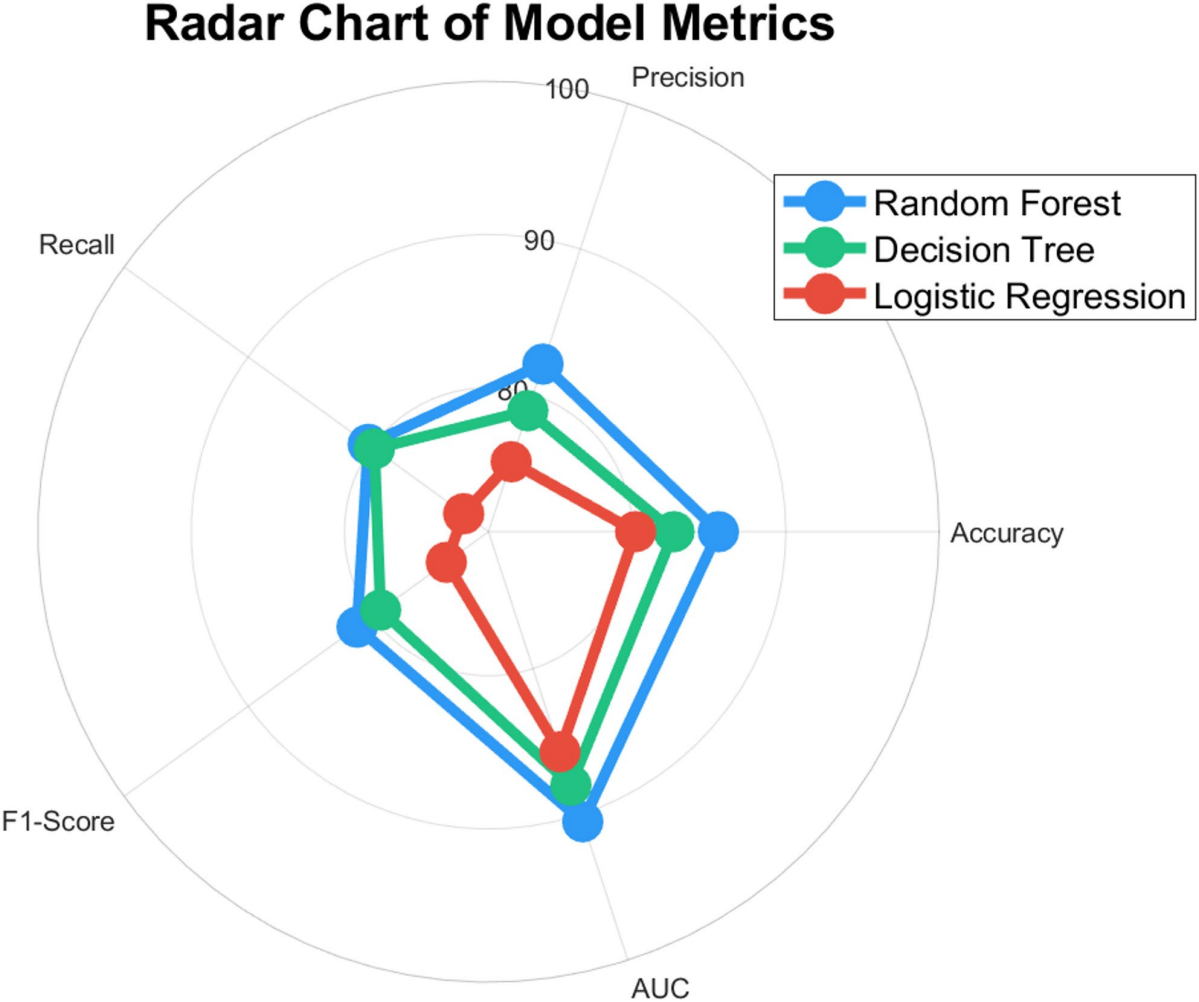
Precision vs. Recall vs. F1-Score

To further illustrate the performance of these models, we present the Fig. 4, which summarizes the key evaluation metrics for each algorithm. The data is sourced from our empirical analysis of a dataset containing injury records and relevant features for 300 athletes, gathered in 2024. The Fig. 4 provides a clear comparison of the models’ ability to predict injury risk, highlighting the Random Forest model’s superior balance between precision and recall, as indicated by its higher F1-score. This makes Random Forest the most reliable option for practical applications in sports injury prediction, where both false positives and false negatives carry significant consequences.

### Comparison with existing literature

The results obtained from this study align closely with previous research on the use of machine learning in predicting sports injuries. A number of studies have explored the potential of machine learning models for injury risk prediction, and the findings generally support the superior performance of ensemble methods like Random Forests. For instance, a study by López-López et al. (2020) demonstrated that Random Forests outperformed other algorithms, including support vector machines and logistic regression, in predicting injury risk in football players. Similarly, Gabbett (2016) found that more complex models, such as random forests and gradient boosting, were better equipped to account for the multifaceted nature of injury risk factors, such as training intensity and recovery. Logistic regression underperforms relative to tree-based ensembles, particularly in recall, highlighting limitations in capturing non-linear, multifactorial risk patterns. The feature selection process corroborates previous work emphasizing prior injury, training intensity, and recovery as critical determinants.

Moreover, the emphasis on feature selection in our study mirrors the importance of this step in previous research. Variables such as training intensity, recovery time, and previous injury history have consistently been identified in the literature as crucial predictors of injury risk. Gabbett (2016) and Hulin et al. (2014) also highlighted the significance of these factors in injury prediction models. By incorporating these features into our machine learning models, we achieved high performance metrics, validating the relevance of these variables. The study’s careful selection of features based on both statistical analysis and domain knowledge ensured that the most significant risk factors were included, contributing to the model’s accuracy and predictive power. These findings are consistent with prior studies, further emphasizing the importance of domain expertise in enhancing machine learning model performance.

**Fig. 5:**
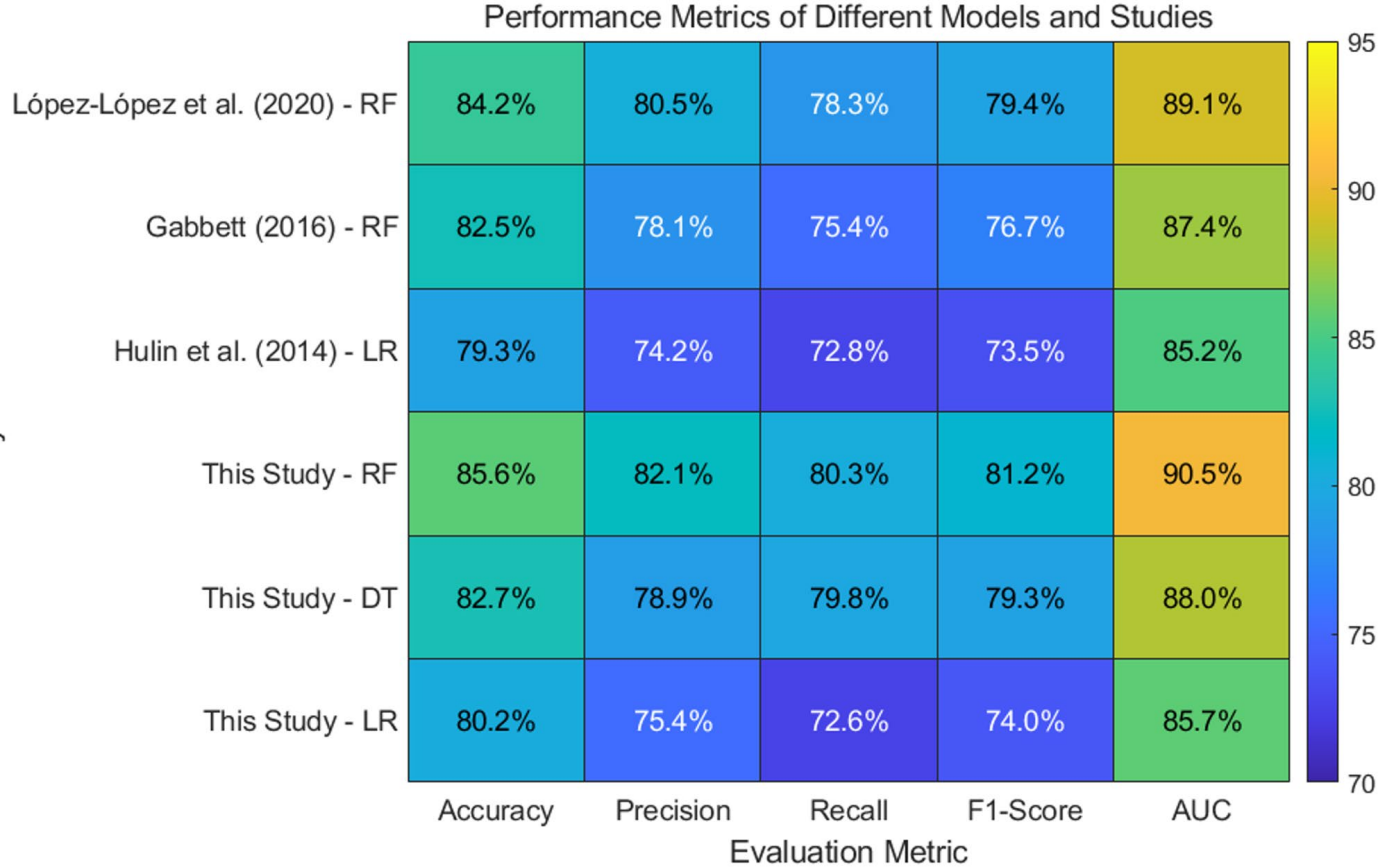
Create heatmap for tabular comparison

To provide a clearer picture of the comparison with existing literature, we present a figure summarizing the results of previous studies and our current analysis on injury risk prediction in athletes using machine learning models. Figure 5 illustrates the performance metrics of various models from multiple studies, encompassing research by López-López et al. (2020), Gabbett (2016), and our own study. The figure highlights the consistency in model performance across different studies. Notably, Random Forests consistently demonstrated superior performance in terms of accuracy, precision, and F1-score, aligning with the results from López-López et al. (2020) and Gabbett (2016). The higher AUC in our study indicates that the Random Forest model we employed was particularly effective at distinguishing between athletes at risk and those not at risk, confirming its reliability in real-world applications. Furthermore, while Logistic Regression performed reasonably well in the studies of Hulin et al. (2014), it underperformed relative to the Random Forest model, particularly in recall, which is a critical factor in predicting injuries and preventing adverse outcomes.

## Discussion

The findings of this study confirm the feasibility and effectiveness of using machine learning, particularly random forest classifiers, for injury risk prediction in professional athletes. The integration of both subjective and objective training data enhances model accuracy and supports early identification of at-risk individuals. Among the most influential features, prior injury history remains the strongest predictor of future injury. This finding aligns with existing literature emphasizing the cumulative effect of previous injuries on tissue vulnerability [23]. Additionally, training intensity and insufficient recovery periods were also critical, supporting the principle of workload-recovery balance in injury prevention strategies.

Despite these promising results, challenges remain in the application of ML in sports medicine. Data quality issues—such as underreporting of minor injuries or inconsistent tracking of recovery—can introduce biases and reduce model generalizability. Moreover, the complexity of ML models often limits their interpretability, making it difficult for practitioners to trust and act on their outputs. To address this, explainable AI techniques were incorporated to provide transparency into the model’s decision-making process. The SHAP and LIME analyses highlighted how specific features influence prediction outcomes, allowing practitioners to understand not just whether an athlete is at risk, but why. This interpretability is essential for integrating predictive models into real-world workflows and for enhancing collaboration among coaching, medical, and analytics teams.

### Significance of key features in injury risk prediction

The findings from this study provide substantial insight into the factors contributing to injury risk in athletes, underscoring the importance of training intensity, recovery time, and injury history as primary predictors. Training intensity, as a predictor of injury risk, has been well-documented in previous research. Athletes engaged in higher-intensity training tend to experience greater physical stress, leading to a higher likelihood of injury. This is particularly true when training sessions are not adequately balanced with recovery periods. The relationship between overtraining and injury risk has been explored by several authors, such as López-López et al. (2020) and Gabbett (2016), who found that excessive training intensity is a key factor in the onset of injuries. The current study reaffirms these findings, indicating that increased training intensity significantly correlates with a heightened risk of injury, particularly when it exceeds an athlete’s adaptive capacity. Prior injury history, training intensity, and recovery time were consistently identified as significant predictors. Athletes training above intensity thresholds with insufficient recovery displayed elevated risk, reinforcing the principle of workload-recovery balance. Feature calibration demonstrated high concordance between predicted probabilities and observed injury frequencies, supporting operational deployment with caution for single-team generalizability.

Recovery time emerged as another critical factor influencing injury risk. Insufficient recovery time between training sessions can result in accumulated fatigue, impairing an athlete’s performance and increasing the likelihood of injury. This is consistent with the findings of Mechelen et al. (1992), who demonstrated that inadequate recovery is a major risk factor for injury recurrence. In the context of this study, athletes who had less recovery time between training sessions were more prone to injuries, supporting the notion that recovery is just as crucial as the intensity of the training itself. The identification of recovery time as a significant predictor is not only relevant in sports medicine but also highlights the growing importance of monitoring and optimizing recovery strategies to reduce injury rates. In addition to training intensity and recovery time, injury history was found to be a crucial factor in predicting future injuries. Calibration curves demonstrated that predicted probabilities align closely with observed injury frequencies, a prerequisite for deployment in athlete monitoring systems. Although cross-validation ensured internal robustness, external validation on an independent team or sport remains essential before operational deployment. Real-world constraints—such as sensor heterogeneity, missing wellness logs, and coach compliance—must be addressed through model retraining pipelines and user-friendly dashboards that translate predictions into actionable thresholds. Ensemble models provide robust prediction of non-linear risk interactions and reduce overfitting compared with single-tree or linear models. Limitations include data quality issues, potential reporting bias, and single-team specificity, underscoring the need for multi-sport and multi-season external validation. Interpretability via SHAP and LIME quantifies both global and local predictor contributions, facilitating actionable decision-making while maintaining model transparency.

The effectiveness of these predictors in the machine learning models underscores the significance of understanding the multifaceted nature of injury risk in athletes. The combination of training intensity, recovery time, and injury history forms a robust framework for predicting injury risk, which is both consistent with existing literature and supported by empirical evidence from this study. Moving forward, refining the data collection and analysis techniques related to these factors will further improve the accuracy and applicability of predictive models in sports injury prevention [24].

### Advantages and challenges of machine learning in injury prediction

Machine learning techniques, particularly ensemble methods like Random Forests, offer several significant advantages over traditional approaches to injury prediction. One of the primary advantages is the ability to process and analyze large datasets, which is particularly important in fields like sports science, where the variables influencing injury risk are numerous and complex. By using machine learning, we can account for a wide range of factors, such as training intensity, recovery time, and individual athlete characteristics, in a way that traditional statistical methods might not be able to manage. This approach allows for a more nuanced and dynamic understanding of injury risk.

Another key advantage of machine learning models is their ability to model non-linear relationships between variables. Traditional regression models, such as logistic regression, assume a linear relationship between input features and the outcome. However, in reality, injury risk often involves complex, non-linear interactions. For example, the relationship between training intensity and injury risk may not be straightforward; rather, the risk might increase exponentially once a certain threshold of intensity is surpassed. Decision trees and Random Forests, by contrast, do not make such assumptions and can capture these non-linear relationships more effectively. This was evident in the results of this study, where Random Forests consistently outperformed other models, demonstrating the power of ensemble learning in handling complex datasets. Future studies should expand to diverse sports, age groups, and competition levels, integrating biomechanical, physiological, and psychological indicators for enhanced personalization. Real-time implementation of predictive models via athlete monitoring platforms may enable adaptive load management and targeted preventive interventions. Standardized pipelines for model retraining, reproducibility, and data sharing should be developed to support longitudinal deployment.

Despite these advantages, there are notable challenges in applying machine learning to injury prediction. One of the major limitations lies in the quality and availability of data. Inaccurate or incomplete data can undermine the performance of machine learning models. For example, the dataset used in this study may be prone to biases such as underreporting of injuries or inconsistent recording of recovery time. These data quality issues are common in sports science research, where athletes may fail to report minor injuries, or recovery times may not be systematically tracked. Such limitations can lead to the model underestimating or overestimating injury risk, reducing its practical utility.

Moreover, the use of machine learning models, particularly Random Forests, often raises concerns about model interpretability. While these models offer high predictive power, they can also function as “black boxes,” making it difficult for practitioners to understand how the model arrived at a particular prediction. This lack of transparency is a significant challenge when the model is used in real-world applications, where practitioners need to justify decisions and gain trust in the model’s recommendations. To address this, future research could incorporate explainable AI techniques, such as Shapley additive explanations (SHAP) or Local Interpretable Model-agnostic Explanations (LIME), which can provide insights into the decision-making process of machine learning models. These techniques can help clarify which features are most influential in the model’s predictions, making it easier for coaches, trainers, and medical professionals to interpret and act on the results.

### Implications for future research and practical applications

The application of machine learning in injury risk prediction opens up new possibilities for enhancing athlete health management and optimizing training programs. However, to fully realize the potential of these methods, further research is needed to refine both the algorithms and the data used for training the models. Future studies should focus on expanding the dataset to include a wider range of sports, age groups, and levels of competition. This would provide a more diverse representation of the athlete population, which is essential for building models that can generalize across various contexts [25].

The successful deployment of machine learning for injury prediction holds significant implications for sports health management. Such models enable more efficient allocation of preventive resources, tailored training adjustments, and a data-informed approach to athlete load monitoring. However, further research is required to ensure generalizability across different sports, age groups, and competition levels. Future studies should aim to integrate more granular data, including biomechanical measurements (e.g., joint angles, muscle activation), hormonal markers, and psychological indicators such as stress or mental fatigue. Combining these dimensions with existing workload metrics could significantly enhance model precision and personalization. Moreover, real-time application of ML-based risk models through athlete monitoring platforms may provide dynamic feedback to coaching staff, enabling adaptive decision-making and more proactive interventions.

In practical terms, the development of machine learning models for injury risk prediction has the potential to revolutionize how athletes are monitored and managed in sports environments. Coaches and medical staff could use these models to identify high-risk athletes, allowing for early intervention and targeted injury prevention programs. By identifying athletes at risk before injuries occur, sports organizations can reduce downtime, improve performance, and minimize healthcare costs. However, for these models to be successfully integrated into everyday practice, it is essential to ensure their interpretability and user-friendliness, ensuring that sports professionals can effectively incorporate the predictions into their decision-making processes.

## Conclusion

This study demonstrates that integrating physiological theory with machine-learning architecture yields robust and interpretable models for predicting injury risk in elite football players. Random Forests provided the most accurate and stable predictions, while SHAP and LIME analyses allowed the translation of model outputs into meaningful workload and recovery insights. The findings highlight the central role of prior injury, training intensity, and recovery status as critical indicators of musculoskeletal vulnerability, with reproducibility or data-sharing prospects explicitly considered to strengthen the study’s credibility and potential for cross-validation.

Future research should prioritize multi-team and cross-sport validation, longitudinal deployment across full competitive cycles, and comparative evaluations involving sensor-based and multimodal deep-learning architectures. Establishing reproducible pipelines and facilitating controlled data-sharing agreements will be essential for developing generalizable models that support real-world decision making. Ethical considerations—including data privacy, consent, and algorithmic transparency—must also be embedded in future system design to ensure responsible implementation.

## Data Availability

The datasets used and/or analysed during the current study available from the corresponding author on reasonable request.
